# Effect of pork carnosine level on lipid and protein oxidation markers during *in vitro* co‐digestion in a Mediterranean meal model

**DOI:** 10.1002/jsfa.70496

**Published:** 2026-02-12

**Authors:** Yi Yao Li, Varoujan Yaylayan, Marie‐France Palin, Tania M. Ngapo, Simon Cliche, Fleur Gagnon, Claude Gariépy

**Affiliations:** ^1^ Department of Food Science and Agricultural Chemistry Macdonald Campus, McGill University Ste Anne de Bellevue Canada; ^2^ Saint‐Hyacinthe Research and Development Centre, Agriculture and Agri‐Food Canada Saint‐Hyacinthe Canada; ^3^ Sherbrooke Research and Development Centre, Agriculture and Agri‐Food Canada Sherbrooke Canada

**Keywords:** antioxidant, dietary carnosine, healthy meal, *in vitro* digestion, meat, pork

## Abstract

**BACKGROUND:**

This study assessed the effects of dietary carnosine during the *in vitro* digestion of a healthy meal model based on the Mediterranean diet, comprising lean pork enriched with two levels of carnosine, whole grain bread, tomato, onion, and olive oil.

**RESULTS:**

Lipid and protein oxidation markers were measured in gastric and duodenal digests, and the total antioxidant capacity was measured in hydrophilic and lipophilic extracts of digests from both phases. Carnosine enrichment of meat increased its bio‐accessibility, simultaneously increasing the total antioxidant capacity and globally reducing oxidation during the digestion of the meal. An increase in malondialdehyde content in gastric digests with an intermediate level of carnosine suggests that, in the absence of significant changes in the ferric‐reducing antioxidant capacity, mechanisms other than the ferric‐reducing capacity of carnosine may account for this pro‐oxidant outcome.

**CONCLUSION:**

Overall, this study confirmed the beneficial properties of carnosine, as a meat constituent, in a healthy meal. © 2026 His Majesty the King in Right of Canada and The Author(s). *Journal of the Science of Food and Agriculture* published by John Wiley & Sons Ltd on behalf of Society of Chemical Industry. Reproduced with the permission of the Minister of Agriculture and Agri‐Food.

## INTRODUCTION

A substantial proportion of the world population is suffering from diet‐induced chronic diseases, such as type 2 diabetes, obesity, and cardiovascular diseases.^1^ In the USA, approximately 60% of adults are estimated to have one or more of these conditions, a major contributing factor being unhealthy eating habits.^2^ Although complex mechanisms are involved in the increased incidence of diet‐related diseases, free radical formation is considered to be critical in this respect, with unhealthy diets providing accelerated oxidation catalyzed by metal ions, generating substrates for oxidation and glycation.^1^, ^3^ In addition to the potential absorption of oxidation products, reactive species can eventually enter the bloodstream, attack target molecules, and increase oxidative stress, interfering with cell signaling and other pathways, and contributing to chronic diseases.^1^, ^3^ Consequently, unhealthy eating habits should be avoided. A healthy diet includes a variety of vegetables and fruits, limited free sugars and salt, and limited and selected sources of carbohydrates and lipids.^4^

According to guidelines and directions for healthy eating, the Mediterranean diet (MD), which includes olive oil as the main source of lipids and abundant plant foods, including whole grains,^5^ is a viable choice.^2^ Numerous studies have confirmed its benefits in preventing chronic diseases.^6^, ^7^ However, studies in this area have focused only on limited aspects, such as the beneficial effects of isolated plant‐origin compounds and the impact of olive oil on oxidation, inflammation, and cancer.^8^, ^9^ Even though meat is an important element of daily diets, and is certainly part of the MD,^10^ bioactive molecules from meat have only received limited attention. For example, Martini *et al*.^11^ reported that plant‐based phenolic compounds exhibit health benefits by reducing hydrogen peroxide and lipid oxidation levels from meat during *in vitro* digestion. In this study,^11^ a Mediterranean vegetable salad, including olive oil, with grilled turkey, was co‐digested. However, carbohydrates, as one of the three major dietary components, were not taken into account and the effect of carnosine, an important functional and beneficial molecule found in meat, was not considered.

Carnosine (*β*‐alanyl‐l‐histidine) is naturally synthesized and abundant in the skeletal muscle of many mammals and is exclusively available in meat, such as beef and pork, and in some fish species.^12^ The chemical, biochemical, and physiological properties of carnosine in supplement (isolated molecule) form have been studied in laboratory settings and in animals and humans, as reviewed by Boldyrev *et al*.^12^ For instance, carnosine possesses pH buffering, metal chelating, antioxidant, antiglycation, and anti‐aging properties.^12^ These properties are manifested in diverse ways, ranging from reducing lipid oxidation during the storage of raw^13^ and cooked meat^14^, ^15^ to improving exercise performance in healthy people and displaying therapeutic potential against many chronic diseases.^16^, ^17^, ^18^

However, the benefits of carnosine reported for isolated compounds, such as supplements, may not fully translate when carnosine is consumed in meat, due to interactions within the meal matrix during digestion. *In vitro* digestion studies of burger‐based meal systems have demonstrated dual roles for carnosine as a meat constituent.^19^, ^20^ In one study, dietary carnosine demonstrated a pro‐oxidative effect in the presence of supplemental ascorbic acid (AA).^19^ In contrast, in another burger‐based meal system, the health‐promoting properties of carnosine in meat were demonstrated irrespective of interactions between carnosine and other anti‐ and pro‐oxidants.^20^ These findings, together with other studies reviewed by Sotler *et al*.,^21^ suggest that the presence and concentrations of bioactive components, including carnosine, AA, phenolics, and other dietary antioxidants, in a meat‐containing MD may all contribute to unexpected pro‐oxidant effects. The digestion process can also create conditions that favor multiple oxidative reactions and interactions.^22^ Under certain circumstances, alterations in this oxidative environment may even cause antioxidants to exhibit pro‐oxidant effects.^21^

Despite these complexities, carnosine in a meat matrix may still provide health benefits in an MD meal. The combined effects of dietary carnosine and other antioxidants present in the same meal during digestion, however, warrant further investigation. One potential outcome is a sparing effect on carnosine during digestion, whereby abundant MD antioxidants may preserve carnosine, increasing its availability for absorption and enhancing its potential *in vivo* health benefits. However, no *in vitro* digestion studies have been performed to ascertain whether such effects occur.

The objective of the current study was, therefore, to determine the accessible level and effect of carnosine in a meat matrix during *in vitro* digestion of a pork‐containing MD model consisting of lean ground pork, tomato, onion, whole grain bread, and olive oil.

## MATERIALS AND METHODS

### Chemicals and reagents

All reagents and chemicals were of analytical grade or higher. Methanol (≥ 99.9%), liquid chromatography–mass spectrometry (LC–MS) grade anhydrous acetonitrile, formic acid (LC–MS grade), 37% hydrochloric acid, and ferrous sulfate heptahydrate were purchased from Thermo Fisher Scientific (Waltham, MA, USA); l‐carnosine‐d_4_, 4‐hydroxyhexenal‐d_3_ (≥ 99%, HHE‐d_3_) and 4‐hydroxynonenal (≥ 99%, 4‐HNE) were obtained from Cayman Chemical Company (Ann Arbor, MI, USA); and *O*‐(2,3,4,5,6‐pentafluorobenzyl)hydroxylamine hydrochloride (OPFB) was obtained from the American Division of the Tokyo Chemical Industry (Portland, OR, USA). All other chemicals and enzymes were purchased from Sigma‐Aldrich (Saint Louis, MO, USA).

### Mediterranean diet model preparation

The MD model used was based on that described by Martini *et al*.,^11^ with modifications. *Longissimus dorsi* muscles were purchased from a commercial pork processing plant in Quebec, Canada (Olymel S.E.C). Lean tissue was ground using a 6 mm plate, and carnosine levels were adjusted by adding 300 or 600 mg carnosine per 100 g meat to the control, which contained an intrinsic carnosine level of 277 mg per 100 g raw pork (LCar), resulting in medium (577 mg carnosine per 100 g raw pork; MCar) and high (877 mg carnosine per 100 g raw pork; HCar) treatments. The meat was transferred to 60 mL polypropylene wide‐mouth screw‐top containers, which were then sealed in individual vacuum bags, cooked in a water bath to a core temperature of 71 °C, and held at this temperature for 5 min. The cooked meat was removed from the containers, portioned (20 g) into bags, vacuum packaged, and stored at −80 °C. Immediately before digestion, the cooked meat was thawed at room temperature for approximately 15 min.

Tomatoes, red onions, cold‐extracted extra virgin olive oil (President's Choice, Loblaw Companies Ltd, Canada), and whole wheat bread (Gadoua Boulangerie Ltée, Laval, QC, Canada) were purchased in a local supermarket in St‐Hyacinthe (Qc, Canada). Just prior to digestion, the bread, onion, and tomato were independently finely ground using a household blender (approximately 10 s). Cooked pork (1.6 g), oil (0.16 mL), ground fresh bread (0.64 g), tomato (3.2 g), and red onion (0.4 g) were added to a 50 mL polypropylene tube and subjected to *in vitro* digestion.

### *In vitro* digestion

The *in vitro* digestion method was adapted from Li *et al*.^23^ and based on the general food digestion method described by Versantvoort *et al*.,^24^ modified for meat digestion by Van Hecke *et al*.^25^ The medium for the saliva, gastric, and duodenal juices was prepared as described by Van Hecke *et al*.^25^ (Table 1). Tubes containing meal samples (6.0 g) were incubated sequentially for 5 min with saliva (6 mL), for 2 h with gastric juice (12 mL), and for 2 h with 1 mol L^−1^ bicarbonate buffer (pH 8.0, 2 mL), duodenal juice (12 mL), and bile (6 mL). Colonic digestion was not carried out. This enzymatic incubation was performed in quadruplicate. Two tubes of digested samples were taken after the gastric phase, and one after the duodenal phase. The digests from each digestion phase were homogenized at 10 000 rpm for 1 min using a Polytron homogenizer (PT‐MR 3100 with a PT 3012/2 T dispersing aggregate; Kinematica AG, Littau, Switzerland), and aliquots (1.5 mL) were transferred to 2 mL conical tubes and stored at −80 °C until chemical analyses were performed.

**Table 1:** Composition of digestion juices (1 L) from Van Hecke *et al*.

Solution	Saliva (1 L)	Gastric juice (1 L)	Duodenal juice^a^ (1 L)	Bile (1 L)
Inorganic solution	0.90 g KCl	2.75 g NaCl	7.01 g NaCl	5.26 g NaCl
	0.20 g KSCN	0.27 g NaH_2_PO_4_	3.39 g NaHCO_3_	5.79 g NaHCO_3_
	0.90 g NaH_2_PO_4_	0.82 g KCl	0.08 g KH_2_PO_4_	0.38 g KCl
	0.57 g Na_2_SO_4_	0.40 g CaCl_2_·2H_2_O	0.56 g KCl	0.15 mL of 37% HCl
	0.30 g NaCl	0.31 g NH_4_Cl	0.05 g MgCl_2_	
	1.69 g NaHCO_3_	6.50 mL HCl (37%)	0.18 mL HCl (37%)	
Organic solution	0.20 g urea	0.09 g urea	0.10 g urea	0.25 g urea
	11.5 mg uric acid	0.02 g glucuronic acid	1.00 g BSA	1.80 g BSA
	25.0 mg mucin	0.33 g glucosamine‐HCl	9.00 g pancreatin	30.0 g bile
	2.50 IU peroxidase	0.65 g glucose	1.50 g lipase	
		17.6 g ascorbic acid		
		11.2 mg BSA		
		2.50 g pepsin		
		3.00 g mucin		
Add to mixture of inorganic + organic solutions	6.90 mg NaNO_2_	10 μL H_2_O_2_ (30%)	0.20 g CaCl_2_·2H_2_O	0.22 g CaCl_2_·2H_2_O
		FeSO_4_·7H_2_O		
pH	6.8 ± 0.2	1.3 ± 0.02	8.1 ± 0.2	8.2 ± 0.2

Abbreviation: BSA, bovine serum albumin.

^a^ Held for 20 min at 37 °C in a water bath before use.

### Free carnosine measurement

Carnosine concentrations in cooked meat and digests were measured by liquid chromatography–tandem mass spectrometry (LC–MS/MS) as described by Li *et al*.,^20^ adapted from Han *et al*.^26^ Carnosine was extracted from cooked meat by mixing water (5 mL) with meat (0.1 g) in a capped 15 mL polypropylene tube, rotating for 20 min (50 turns per min), and collecting the liquid phase. Extraction was not required for the digests; the gastric digests were diluted tenfold with water, and duodenal digests were diluted fivefold. Meat extract (200 μL) or diluted digest (200 μL) was mixed with l‐carnosine‐d_4_ (200 μL, 50 ng μL^−1^) and water (600 μL). The mixture was passed through an Oasis HLB solid‐phase extraction column (Waters Corporation, Milford, MA, USA) previously conditioned according to the manufacturer's instructions. The extract was filtered through a 0.22 μm nylon membrane syringe filter.

The sample (0.3 mL) was diluted with acetonitrile (0.7 mL), and 5 μL was injected into a Vanquish high‐performance liquid chromatography (HPLC) system coupled with a Linear Trap Quadrupole ‐ Extended Performance (LTQ XL) mass spectrometer equipped with a heated electrospray ionization (HESI‐II) probe (Thermo Fisher Scientific) and fitted with an Acquity ultra‐performance liquid chromatography bridged ethyl hybrid (UPLC BEH) amide column (2.1 × 100 mm, 1.7 μm) (Waters Corporation). Mobile phases consisted of solvent A comprising 10 mmol L^−1^ ammonium acetate (0.1% ammonium hydroxide) in water/acetonitrile (20:80, v/v) and solvent B comprising 10 mmol L^−1^ ammonium acetate (0.1% ammonium hydroxide) in water/acetonitrile (70:30, v/v). Elution was performed with a gradient of 0% B initially; 50% B at 3 min; 80% B at 7 min; 0% B at 13.5 min, followed by equilibration for 6.5 min at a constant flow rate of 0.25 mL min^−1^. The column temperature was maintained at 35 °C, the HESI‐II probe at 275 °C, and the capillary at 300 °C. A spray voltage of 4 kV was applied, with sheath and auxiliary gas settings of 30 and 5 arbitrary units, respectively. Protonated molecular ions were detected at *m/z* 227 for carnosine and *m/z* 231 for carnosine‐d₄.

### Protein oxidation markers (carbonyls and free thiols)

Protein carbonyls were determined using a 2,4‐dinitrophenylhydrazine (DNPH) method as described by Ventanas *et al*.^27^ and modified by Li *et al*.^19^ Briefly, digests (200 μL) were mixed with 20% trichloroacetic acid (TCA, 200 μL) and centrifuged at 3000 × *g* for 5 min. One pellet was treated with 2 mol L^−1^ HCl (2 mL) to measure protein concentration; this served as a blank. The DNPH solution (2 mL of 10 mmol L^−1^ DNPH in 2 mol L^−1^ HCl) was mixed with the other pellet to determine the carbonyl concentration. Both samples were incubated at room temperature for 1 h, after which 20% TCA (2 mL) was added to each, forming a precipitate. The precipitate was washed three times with freshly prepared ethyl acetate:ethanol (1:1, v/v; 2 mL), and fully dissolved in 6 mol L^−1^ guanidine hydrochloride in a 20 mM sodium phosphate buffer (pH 6.5, 2.5 mL). Insoluble fragments, if present, were removed by centrifugation at 2240 × *g* for 2 min. Protein concentration was determined from absorption at 280 nm using bovine serum albumin (BSA) as the standard. Carbonyls were measured at 365 nm using an absorption coefficient of 22 000 L mol⁻¹ cm⁻¹ and expressed as nmol mg⁻¹ protein.

Free thiols were measured using a modified 2, 2′‐dithiobis(5‐nitropyridine) (DTNP) method based on Martinaud *et al*.^28^ Protein concentrations in digests were determined by the biuret method.^29^ The protein content of the digests was adjusted to 5 mg mL^−1^ with 200 mM phosphate buffer (pH 7.4) and further adjusted to 1 mg mL^−1^ with urea buffer (8 M urea in 100 mmol L^−1^ phosphate buffer, pH 8.0). The samples (2 mL) were then mixed with 10 mM DTNP in ethanol (20 μL) and incubated for 1 h at room temperature. Absorbance was measured at 386 nm against a blank of protein at the same concentration without DTNP. The absorbance of diluted DTNP was subtracted from the sample absorbance, and the thiol concentration was calculated using an absorption coefficient^28^ of 14 L mol^−1^ cm^−1^. Results are expressed as μmol free thiol mg⁻¹ protein.

### Lipid oxidation markers

Lipid oxidation products, including hexanal, 4‐hydroxynonenal (4‐HNE), and malondialdehyde (MDA) were measured using a GC–MS method adapted by Li *et al*.^23^ from Tsikas *et al*.^30^ The system comprised an Agilent 7890B gas chromatograph coupled to a 5977B quadrupole mass spectrometer (Agilent Technologies, Santa Clara, CA, USA) with Ultimate Plus deactivated fused silica tubing (5 m × 0.25 mm) and HP‐5MS columns (30 m × 0.25 mm × 0.25 μm).

Briefly, digests (200 μL) were mixed (vortexed for 30 s) with OPFB (170 μL of a 30 mg mL^−1^ solution) and HHE‐d_3_ (10 μL of a 20 ng μL^−1^ solution in ethanol), then sonicated for 3 min in an ultrasonic bath. Extraction of the targeted components was undertaken by the sequential addition of methanol (100 μL), isooctane (1 mL), and concentrated sulfuric acid (six drops), after which the mixture was vortexed for 1 min and centrifuged at 774 × *g* for 5 min. The supernatant (800 μL) was transferred to vials through anhydrous sodium sulfate and glass wool and evaporated to dryness. Samples were dissolved in *N, O*‐bis(trimethylsilyl)trifluoroacetamide (BSTFA, 50 μL) and incubated at 80 °C for 1 h.

The GC–MS analysis was undertaken as described by Li *et al*.,^19^ with changes in the selected ions. Prepared samples (1 μL) were introduced into the instrument and selected‐ion monitoring (SIM) was used. For quantification, the following ions were chosen: 187 and 203 *m/z* (retention time (RT) of 12.20 and 12.33 min for the internal standard HHE‐d_3_); 239 *m/z* (RT 10.35 min for hexanal); 352 *m/z* (RT 13.62 min for 4‐HNE); and 250 m/z (RT 13.76 and 13.79 min for MDA).

### Total antioxidant capacity

Hydrophilic and lipophilic antioxidant extracts were obtained using the method described by Rodríguez‐Roque^31^ with modified solvent volumes. Gastric digest (1.5 mL) and methanol (1.5 mL) were mixed and the methanol layer was retained as the hydrophilic extract. After removing this hydrophilic layer, the remainder was mixed with tetrahydrofuran (1 mL) to obtain the lipophilic extract. The duodenal digest (1.5 mL) was treated with methanol (1 mL) to obtain the hydrophilic extract and tetrahydrofuran (1.5 mL) to obtain the lipophilic extract.

The working solutions and detailed determination steps for each of the assays – 2,2‐diphenyl‐1‐picrylhydrazyl (DPPH), 2,2′‐azino‐bis(3‐ethylbenzothiazoline‐6‐sulfonic acid) (ABTS), and ferric reducing antioxidant power (FRAP) – were prepared as described by Rodríguez‐Roque^31^ and Xiao *et al*.^32^ Briefly, a concentrated DPPH methanol solution (0.5 mg mL^−1^) was prepared and then diluted with methanol to achieve an absorbance of 0.7–0.8 at 515 nm as a working solution. Solvent blank (methanol) or digest extract (0.2 mL) was added to DPPH working solution (1.8 mL) and held for 30 min at room temperature. Absorbances of the blank and samples were obtained at 515 nm, and the inhibition rate was calculated based on the decreased absorbance (the difference between the blank and sample) divided by the absorbance of the blank.

An ABTS mother solution, containing 3.5 mmol L^−1^ of ABTS and 1.225 mmol L^−1^ of potassium persulfate, was prepared with acetate buffer (pH 4.5) and stored in dark conditions at room temperature for 12–16 h (overnight). An ABTS working solution was then obtained by diluting the mother solution with acetate buffer (pH 4.5) to achieve an absorbance of 0.737–0.743 at 734 nm.^32^ Digest extract (10 μL) and working solution (200 μL) were mixed and held in dark conditions at room temperature for 7 min, after which absorbance was obtained at 734 nm.

A working solution of FRAP, containing both 9.09 mmol L^−1^ of 2,4,6‐tri(2‐pyridyl)‐1,3,5‐triazine (TPTZ) and 1.82 mmol L^−1^ of FeCl3, was prepared as described by Xiao *et al*.^32^ and warmed to 37 °C for 1 h. The FRAP working solution (180 μL) was mixed with the digest extract (5 μL) and incubated in dark conditions at 37 °C. The absorbance at 593 nm was read after a 15 min incubation period.

For all three techniques (DPPH, ABTS, and FRAP), Trolox equivalent standard curves were applied to the measurements.

### Statistical analyses

Statistical analyses of all data were carried out using analysis of variance (ANOVA) with SAS version 9.4 (SAS, 2002–2012; SAS Institute Inc., Cary, NC, USA) at three carnosine levels, with the entire protocol being repeated four times. The significance level was *P* ≤ 0.05. The results are presented as least squares means (LS means) with standard errors of the means (SEMs).

## RESULTS AND DISCUSSION

### Carnosine levels during digestion of the healthy meal model

The average intrinsic carnosine level (LCar) in cooked pork was 465.63 ± 37.17 mg 100 g⁻¹ meat. Before digestion, carnosine enhancement in MCar significantly increased the level of carnosine to 710.57 ± 37.17 mg 100 g⁻¹ meat (*P* < 0.0001), and further carnosine enhancement in the HCar group increased its level to 1009.58 ± 37.17 mg 100 g⁻¹ meat, which was significantly (*P* < 0.0001) higher than the MCar and LCar groups.

Carnosine enrichment was also observed in the digests from both phases. In the gastric phase, the carnosine level in MCar digests was increased significantly to 566.15 μg mL^−1^ (*P* = 0.0003) compared with the LCar digests (365.06 μg mL^−1^). The level of carnosine in the HCar digests also reached 744.44 μg mL^−1^, which was significantly higher than that found in both LCar (*P* < 0.0001) and MCar (*P* = 0.0006) digests. In the duodenal phase, the MCar group still presented higher concentrations of free carnosine (286.51 μg mL^−1^) compared with the LCar group (201.52 μg mL^−1^; *P* < 0.0001). The highest concentrations of free carnosine were observed in the HCar treatment (400.51 μg mL^−1^) as compared with the LCar (*P* < 0.0001) and the MCar group (*P* < 0.0001). Irrespective of the treatments, there was a decrease in the level of carnosine from the gastric to the duodenal phase, as previously reported by Li *et al*.^19^, ^23^ using the same digestion protocol. This decrease may be related to the involvement of carnosine in the diminution of oxidation occurring in the gastrointestinal tract (GIT).

Progressive and significant increases in free carnosine concentrations from LCar to MCar to HCar in both digests suggest potential benefits of carnosine‐enhanced meat in the GIT, given carnosine's antiproliferative effect on gastric and colorectal cancer cells.^33^, ^34^ Considering the documented *in vivo* health advantages of carnosine,^12^ the more free carnosine remains in the duodenal digests and becomes accessible for absorption, the more benefits carnosine‐enhanced meat may bring to consumers. Accordingly, the higher levels of bio‐accessible carnosine in MCar and HCar compared with LCar demonstrate the *in vivo* potential of carnosine enrichment in meat. The MD‐based healthy meal system used in the current study also led to more than 90% retention of enriched carnosine in meat after duodenal digestion, compared with about 64% retention in the burger‐based meal system of Li *et al*.^20^ This difference emphasizes potential health benefits of an MD‐based meal as a result of preserving more carnosine for its *in vivo* benefits.

### Lipid and protein oxidation‐related indicators

As Table 2 shows, carnosine treatments had limited effect on the concentrations of protein oxidation markers (protein carbonyls and free thiols). The only significant difference was found in the duodenal phase, where the HCar treatment increased free thiol levels compared with the MCar (*P* < 0.0001) and LCar (*P* < 0.0001) groups. This finding aligns with the beneficial antioxidant potential of carnosine reported by Li *et al*.^23^ during *in vitro* digestion of carnosine‐enriched pork across varying fat levels and cooking methods.

**Table 2:** Effect of carnosine‐enriching treatments on lipid and protein oxidation markers during *in vitro* digestion of a healthy pork‐containing meal^a^

		Carnosine treatment				
Oxidation marker	Digestion phase	Low (LCar)	Medium (MCar)	High (HCar)	SEM	*p* Value
Hexanal (ng mL^−1^ digest)	Gastric	1590^ab^	1891^a^	1312^b^	83.9	**0.0029**
	Duodenal	1050	1071	1045	34.7	0.8479
4‐Hydroxynonenal (4‐HNE) (ng mL^−1^ digest)	Gastric	128^ab^	152^a^	101^b^	9.7	**0.0142**
	Duodenal^b^	‐	‐	‐		
Malondialdehyde (MDA) (ng mL^−1^ digest)	Gastric	245^b^	300^a^	172^c^	11.6	**0.0001**
	Duodenal	135	137	124	8.8	0.5444
Protein carbonyls (nmol mg^−1^ protein)	Gastric	5.31	5.00	4.74	0.19	0.1700
	Duodenal	3.62	3.52	3.71	0.20	0.8205
Thiols (nmol mg^−1^ protein)	Gastric	5.08	5.09	5.36	0.27	0.7198
	Duodenal	2.58^b^	2.25^b^	4.22^a^	0.15	**<0.0001**

^a^ Values with different letters in the same row differ significantly (*P* ≤ 0.05).

^b^ Not quantified.

In contrast to protein oxidation markers, carnosine enrichment of meat significantly affected lipid oxidation products in the gastric digests, with no effects observed in the duodenal phase. In the gastric phase, MCar treatment did not alter hexanal or 4‐HNE concentrations compared with LCar. However, HCar digests showed decreased levels of hexanal (*P* = 0.0022) and 4‐HNE (*P* = 0.0112) relative to MCar, supporting the antioxidant role of carnosine in various environments, including during digestion.^12^, ^23^

In gastric digests, MDA unexpectedly increased in the MCar treatment (300.56 μg mL⁻¹) compared with LCar (244.53 μg mL⁻¹; *P* = 0.0195). This may reflect the pro‐oxidative potential of carnosine, as observed by Li *et al*.^19^ in an *in vitro* digestion study of a burger meal model in the presence of AA at a supplemental level. In the HCar gastric digests, however, MDA concentration was significantly lower than in both MCar (*P* = 0.0195) and LCar (*P* < 0.0001) digests, indicating an antioxidant property of carnosine at the higher concentration in the current study. Although MDA was not measured in the Li *et al*. study,^19^ a potential pro‐oxidant effect of carnosine at the MCar level was observed in the measurement of other markers. For instance, in the presence of a high level of AA, the increased level of carnosine in meat significantly promoted oxidation and led to more 4‐HNE at the MCar level during digestion of the meal, but further increasing carnosine in the HCar group significantly reduced the level of 4‐HNE compared to the MCar treatment. The most likely explanation for the dual effects of carnosine in the context of Li *et al*.,^19^ and particularly the pro‐oxidative effect, can be the ferric‐reducing capacity of both carnosine and AA contributing to the regeneration of ferrous ions in the presence of hydroperoxide, subsequently leading to the initiation of lipid oxidation.

Overall, irrespective of the carnosine treatments, hexanal and MDA decreased from the gastric phase to the duodenal phase, with 4‐HNE decreasing to a level below the quantification limit in the latter. These decreases are not isolated observations; similar trends were also noticed across meal models throughout digestion. Li *et al*.^23^ reported decreases in 4‐HNE and MDA levels from gastric to duodenal phases in a study on *in vitro* digestion of carnosine‐enhanced pork. Studies on the *in vitro* digestion of different burger meal models^19^, ^20^ also reported similar decreases in 4‐HNE levels during digestion (Table 3). These decreases could be attributed to dietary antioxidants (including carnosine) being released during digestion and exerting their antioxidant effects. The ability of carnosine to bind directly to 4‐HNE, a process that could be enhanced under the relatively basic environment of the duodenal phase,^20^ may also contribute. Lower concentrations of lipid oxidation products were also found in the current study employing an MD‐based healthy diet model than previously reported for the other two burger‐based meal systems.^19^, ^20^ For instance, hexanal levels in the duodenal digests of the current study were approximately 50% of those in the burger meal combo^20^ and about 10% of those in the burger meal model with supplemental ascorbic acid.^19^ Similarly, MDA levels in duodenal digests of the current study were lower (approximately 20%) than those detected in the burger meal model.^20^ Despite differences in meal composition, the consistency in digestion protocol and method for lipid oxidation measurement allows for a reliable comparison of oxidation outcomes across different meal studies, including the current study. Considering the potential intestinal absorption of these lipid oxidation products and their health implications,^35^ the lower levels of oxidation products observed in duodenal digests of the current MD‐based model underscore its suitability as a healthy diet system and demonstrate the value of a healthy diet.

**Table 3:** Key information from studies of the effect of dietary carnosine on the *in vitro* digestion of meat and meat‐containing meals

		Burger meal model including ascorbic acid (AA) at a supplemental level			Burger meal model including AA at a dietary level	Cooked meat of varied fat content and cooking conditions	Comments
		*LCar* ^a^	*MCar*	*HCar*			
Carnosine (μg mL^−1^)	Gastric	283 ± 222	421 ± 382	529 ± 465	920 ± 218	1121 ± 140	Decreased carnosine during digestion regardless of treatments.
		144 ± 51	202 ± 70	317 ± 135	513 ± 127	707 ± 206	
	Duodenal						
4‐Hydroxynonenal (4‐HNE) (ng mL^−1^)	Gastric	248 ± 71	302 ± 23	291 ± 50	90 ± 50	127 ± 95	Decreased 4‐HNE during digestion regardless of treatments.
		83 ± 10	111 ± 24	107 ± 44	Below the limit of quantification	81 ± 69	
	Duodenal						
							Pro‐oxidant effect of carnosine in reference.^19^
Hexanal (μg mL^−1^)	Gastric	5.00 ± 0.42	6.13 ± 0.88	5.49 ± 0.72	1.66 ± 0.27	3.96 ± 2.19	Increased hexanal during digestion regardless of treatments.
		10.34 ± 1.15	15.76 ± 5.45	16.84 ± 7.53	2.34 ± 0.54	5.11 ± 3.63	
	Duodenal						
							Pro‐oxidant effect of carnosine in reference.^19^
Malondialdehyde (MDA) (ng mL^−1^)	Gastric	‐	‐	‐	583 ± 125	3408 ± 1641	
	Duodenal						
		‐	‐	‐	679 ± 186	3188 ± 1584	
Reference number			19		20	23	

^a^ LCar, low carnosine level; MCar, medium carnosine level; HCar, high carnosine level.

### Total antioxidant capacity

The total antioxidant capacity (TAC) was measured for direct information on the potential benefits brought about by different levels of carnosine during digestion. Carnosine is hydrophilic, and the Mediterranean diet provides antioxidants that are both hydrophilic, such as ascorbic acid and phenolic compounds, and lipophilic, such as carotenoids.^11^ Hydrophilic and lipophilic antioxidant extracts are necessary for a comprehensive assessment of the total antioxidant capacity of digests. It is also recommended in the literature that a combination of different assays should be employed for more comprehensive determination when analyzing a complex food‐based matrix.^36^ Given that hydrogen atom transfer (HAT) and electron transfer (ET) are the two major mechanisms underlying the TAC assays, widely used methods based on mixed mechanisms^36^ were chosen in the current study. The DPPH technique relies more on the ET, whereas the ABTS assay relies more on the HAT mechanism.^37^ Both DPPH and ABTS methods can also target radical scavenging ability, which is a well‐known property of carnosine.^12^, ^36^ The FRAP assay was used because the ferric‐reducing ability of carnosine and ascorbic acid can contribute to their pro‐oxidant potential.^19^, ^38^, ^39^ The DPPH, ABTS, and FRAP assays were all used for the determination of both hydrophilic and lipophilic extracts of gastric and duodenal digests.^11^, ^31^, ^36^

**Figure 1:**
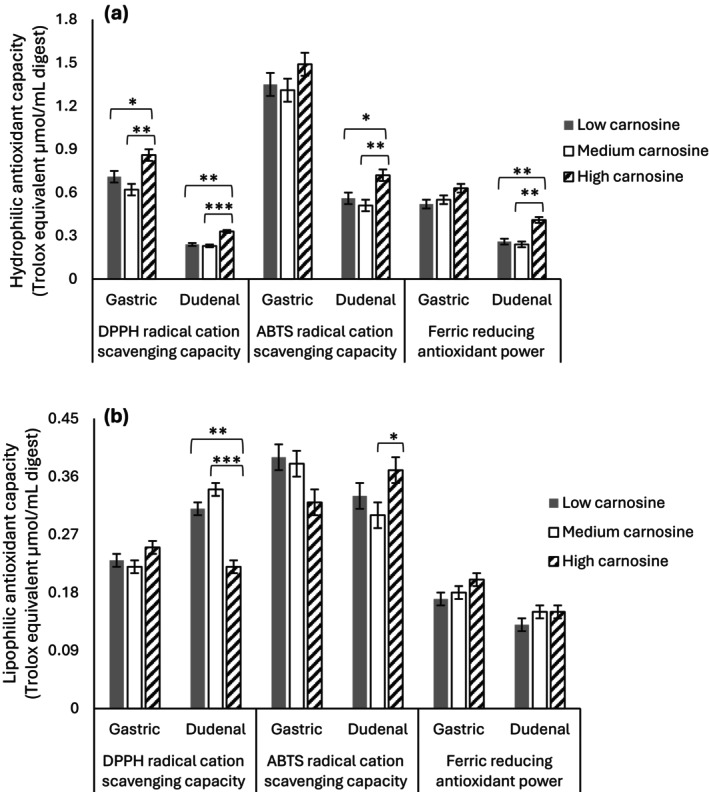
Effect of carnosine‐enriching treatments on (a) hydrophilic and (b) lipophilic antioxidant capacity during *in vitro* digestion of a healthy meal containing pork. *P < 0.05; **P < 0.01; ***P < 0.001; ****P < 0.0001. DPPH, 1,1‐diphenyl‐2‐picrylhydrazyl; ABTS, 2,2′‐azino‐bis(3‐ethylbenzothiazoline‐6‐sulfonic acid).

Table 4 and Fig. 1 show TAC in each gastric and duodenal digest phase. Using the DPPH method, TAC in hydrophilic extracts was significantly higher in HCar for both gastric and duodenal digests, consistent with the antioxidant activity of carnosine, which is highly water soluble (Fig. 1(a)). Carnosine has been reported to react with DPPH radicals,^40^ and this histidine‐containing peptide could act as an antioxidant primarily through ET‐based pathways.^41^ No significant differences were observed in the lipophilic extracts of gastric digests, whereas HCar lipophilic extracts showed significantly lower TAC in the duodenal phase (Fig. 1(b)).

**Table 4:** Effect of carnosine‐enriching treatments on total antioxidant capacity during *in vitro* digestion of a pork‐containing healthy meal^a^

		DPPH^c^ radical scavenging capacity (Trolox equivalent μmol mL^−1^ digest)		ABTS^d^ radical scavenging capacity (Trolox equivalent μmol mL^−1^ digest)		Ferric reducing antioxidant power (FRAP) (Trolox equivalent μmol mL^−1^ digest)	
Extract	Carnosine level^b^	Gastric	Duodenal	Gastric	Duodenal	Gastric	Duodenal
Hydrophilic	LCar	0.71^b^	0.24^b^	1.35	0.56^b^	0.52	0.26^b^
	MCar	0.62^b^	0.23^b^	1.31	0.51^b^	0.55	0.24^b^
	HCar	0.86^a^	0.33^a^	1.49	0.72^a^	0.63	0.41^a^
SEM		0.04	0.01	0.08	0.04	0.03	0.02
*P* values		**0.0040**	**0.0005**	0.2860	**0.0078**	0.1334	**0.0009**
Lipophilic	LCar	0.23	0.31^a^	0.39	0.33^ab^	0.17	0.13
	MCar	0.22	0.34^a^	0.38	0.30^b^	0.18	0.15
	HCar	0.25	0.22^b^	0.32	0.37^a^	0.20	0.15
SEM		0.01	0.01	0.02	0.02	0.01	0.01
*P* values		0.2322	**0.0003**	0.1141	**0.0486**	0.0835	0.5688

^a^ Different superscript letters in the same column and within the same type of extract differ significantly (*P* ≤ 0.05).

^b^ LCar, low carnosine level; MCar, medium carnosine level; HCar, high carnosine level.

^c^ DPPH: 1,1‐Diphenyl‐2‐picrylhydrazyl.

^d^ ABTS: 2,2′‐Azino‐bis(3‐ethylbenzothiazoline‐6‐sulfonic acid).

The ABTS assay showed changes brought about by carnosine, not only in the antioxidant ability of the hydrophilic extracts, but also in the lipophilic extracts. Carnosine treatments had no effect in the gastric digests. However, significantly higher levels of TAC in the HCar group were observed compared with the LCar and MCar groups in the hydrophilic duodenal extracts, and with the MCar group in the lipophilic duodenal extracts (Fig. 1). Even though carnosine is not a lipophilic antioxidant, it is possible that carnosine exerted its antioxidant activity at the water–lipid interface, subsequently contributing to interruption of the chain reactions of lipid oxidation in the lipid phase.^42^ The ABTS technique, which involves both HAT and ET mechanisms, can also better assess the antioxidant capacity of HAT‐based antioxidants than the other two techniques, which rely more on the ET mechanism (DPPH and FRAP).^36^ Accordingly, higher TAC levels were reported by the ABTS technique compared with the other two TAC methods used in the current study. Considering histidine's ability to donate protons, it is also plausible that carnosine as a histidine‐containing peptide might contribute to higher levels of TAC through the HAT mechanism. However, more studies are needed to validate this possibility and determine the extent to which this mechanism could contribute.

The FRAP method is only based on the ET mechanism and focuses on the ferric‐reducing ability of the antioxidant. No significant changes in TAC levels determined by this method were observed in gastric digests. In the duodenal phase, no significant differences were observed in lipophilic extracts, whereas the TAC determination for the hydrophilic extracts showed significantly higher antioxidant capacity/ferric‐reducing capacity (*P* = 0.0009) of the HCar group compared with the MCar and LCar groups (Fig. 1(a)). This observation in duodenal digests is consistent with the antioxidant effect of carnosine and supports the beneficial property of this dipeptide in the meat matrix when co‐digested with an antioxidant‐rich meal.

The lack of significant changes in MCar gastric digests found by the FRAP method does not support the hypothesis that ferric‐reducing capacity contributed to the pro‐oxidant effect of carnosine on MDA levels. The carnosine treatment also caused no changes in hydroperoxide concentrations in either phase (data not shown). Given the same pork matrices^20^ and digestion conditions in the experiments using burger and healthy meal models, these observations indicate the complexity of conditions in healthy meal digests, where the redox cycle between ferrous and ferric ions could contribute to Fenton‐like reactions, but might not be the only acting mechanism. For instance, phenolic compounds and carotenoids present in the meal containing onions and tomatoes can all have pro‐oxidative potentials through different mechanisms.^11^, ^43^, ^44^, ^45^ When acting as antioxidants, carotenoids scavenge free radicals and can form reactive carotenoid radicals, contributing to the overall oxidation process.^45^ The pro‐oxidative effect of phenolic compounds can also be related to benzoquinone formation, which is unrelated to ferric‐reducing ability.^43^ These bioactive compounds may also interact with each other. For instance, ascorbic acid (AA) can eliminate the very reactive carotenoid radical cations formed when radicals are scavenged by carotenoids showing antioxidant activity,^45^ reducing the pro‐oxidative potential of carotenoids, while AA consumption may limit its participation in other reactions, such as promoting lipid peroxidation.^46^ However, these complex interactions among phytonutrients and the interaction of dietary carnosine with other bioactive compounds warrant further investigation.

## CONCLUSION

Carnosine‐enriched meat in a healthy meal reduced oxidation levels and increased total antioxidant capacity during gastrointestinal digestion. Compared with burger meal models reported by Li *et al*.,^19^, ^20^ in which the antioxidant activity of carnosine occurred at the expense of its bio‐accessibility, the lower oxidative environment provided by the healthy meal preserved some carnosine from acting as an antioxidant, increasing the level of free carnosine for absorption and potential further *in vivo* benefits. In the gastric phase, a high carnosine level (HCar) decreased lipid oxidation and increased the antioxidant capacity in the lipid environment. This HCar treatment also controlled protein oxidation and increased antioxidant capacity in both aqueous and lipid environments in the duodenal phase. By contrast, medium carnosine levels (MCar) promoted MDA formation in the gastric digests, and HCar decreased the antioxidant ability of lipophilic extracts against the DPPH radical in the duodenal phase. Together, these results suggested that carnosine displayed a pro‐oxidant potential under certain oxidative conditions, as previously observed by Li *et al*.^19^, ^20^ with burger meal models. The combination of elevated MDA levels and unchanged FRAP observed in MCar gastric digests suggests that the ferric‐reducing capacity of carnosine contributing to Fenton reactions may not be the only mechanism involved in accelerating lipid oxidation in the current study. Further studies are therefore required to understand how the carnosine level, in the presence of other antioxidants, can promote MDA formation.

The benefits of carnosine in a healthy meal represent an incentive to produce carnosine‐rich meat, which can be achieved by feeding practices, for example through increased levels of l‐histidine in pig feed.^47^

## CONFLICT OF INTEREST

The authors declare no conflicts of interest.

## Data Availability

The data that support the findings of this study are available from the corresponding author upon reasonable request.
